# Fuzzy Logic-Based Control for a Morphing Wing Tip Actuation System: Design, Numerical Simulation, and Wind Tunnel Experimental Testing

**DOI:** 10.3390/biomimetics4040065

**Published:** 2019-09-21

**Authors:** Shehryar Khan, Teodor Lucian Grigorie, Ruxandra Mihaela Botez, Mahmoud Mamou, Youssef Mébarki

**Affiliations:** 1 École de Technologie Supérieure, Laboratory of Active Controls, Avionics and AeroServoElasticity LARCASE, Montreal, QC H3C-1K3, Canada; sherykhann@yahoo.com (S.K.); Ruxandra.Botez@etsmtl.ca (R.M.B.); 2 Military Technical Academy “Ferdinand I”, Faculty of Aircraft and Military Vehicles, Center of Excellence in Self-Propelled Systems and Technologies for Defense and Security, Bucharest 040531, Romania; 3 Aerodynamics Laboratory, NRC Aerospace, National Research Council Canada, Ottawa, ON K1A0R6, Canada; Mahmoud.Mamou@nrc-cnrc.gc.ca (M.M.); Youssef.Mebarki@nrc-cnrc.gc.ca (Y.M.)

**Keywords:** morphing wing, experimental model, control system, fuzzy logic, BLDC motors, wind tunnel testing, pressure data processing, infrared thermography

## Abstract

The paper presents the design, numerical simulation, and wind tunnel experimental testing of a fuzzy logic-based control system for a new morphing wing actuation system equipped with Brushless DC (BLDC) motors, under the framework of an international project between Canada and Italy. Morphing wing is a prime concern of the aviation industry and, due to the promising results, it can improve fuel optimization. In this idea, a major international morphing wing project has been carried out by our university team from Canada, in collaboration with industrial, research, and university entities from our country, but also from Italy, by using a full-scaled portion of a real aircraft wing equipped with an aileron. The target was to conceive, manufacture, and test an experimental wing model able to be morphed in a controlled manner and to provide in this way an extension of the laminar airflow region over its upper surface, producing a drag reduction with direct impact on the fuel consumption economy. The work presented in the paper aims to describe how the experimental model has been developed, controlled, and tested, to prove the feasibility of the morphing wing technology for the next generation of aircraft.

## 1. Introduction

Rising environmental concerns, fuel prices, and ever-increasing operational costs have raised the concerns of aviation industry. Over the last two decades, the industry has increased its collaboration with academia and research institutes around the world in an effort to accelerate the technological advancements of aircraft systems with an ultimate aim of reducing the overall operating cost. On these lines several initiatives were started by industry around the world.

Among these research initiatives, the NOVEMOR project targeted the development of new concepts related to structures, aerodynamics, aeroelasticity, adaptive morphing wing, noise impact reduction, and loads reduction [1]. A similar initiative is SARISTU, which was launched with the objectives of fuel optimization and consequently low passenger fares [2]. In another collaboration between academia and industry, the FutureWings project-funded research objective was to develop a wing with the ability to change its shape by using self-using piezoelectric fibers embedded into composite materials [3]. The LeaTop project focused on the development of Leading Edge Actuation Topology and identified important bottlenecks in the design of leading edge morphing actuation [4]. FlexSys, in its collaboration with Air Force Research Laboratory, developed variable geometry trailing edge structures, which were composed of three wind tunnel models and a flight test specimen [5].

Within the Joint European Initiative on Green Regional Aircraft frame, the researchers from CIRA in cooperation with the University of Naples, Department of Aerospace Engineering, used the Shape Memory Alloy (SMA) technology in the development of morphing wing architectures [6,7]. Morphing wings are in demand because they can reduce the drag on the aircraft wings and which results in fuel optimization. Additionally, they can reduce the weight of the aircraft by eliminating the need for conventional flaps and ailerons. At Texas A&M University-Kingsville, a morphing wing was developed by using elastomeric composites as skins and actuators [8]. Various studies related to this trend were performed also in Military Technical Academy in Romania [9,10,11,12]. A review related to the development of pneumatic artificial muscles has been realized by the American researchers from the University of Maryland [13]; the target was to highlight theirs applications in the morphing wing field. DARPA, in collaboration with NASA and AFRL, completed a morphing wing project where the objectives were to replace the conventional control surfaces with the hinge less morphing variable geometry control surfaces [14]. An international collaborative team, with researchers from Politecnico di Torino, Italy, and RMIT University, Australia, realized the design, analysis, and experimental testing of a morphing wing with the aim of obtaining a new wing concept that is unaffected by aerodynamic losses due to geometrical discontinuity typical of wing-flap assembly [15]. The Indian Institute of Technology performed also some structural and aerodynamics studies on various wing configurations for morphing [16]. A collaboration between the University of Tokyo and the Japan Aerospace Exploration Agency was concretized in the design, manufacturing, and wind tunnel testing of a of variable camber morphing airfoil using corrugated structures [17]. A research team from ONERA in France studied morphing winglet concepts with the aim to improve the load control and the aeroelastic behavior of civil transport aircraft [18].

In this international context, our team, from the Research Laboratory in Active Controls, Avionics and Aeroservoelasticity (LARCASE), Ecole de Technologie Supérieure (ETS) in Montréal, Canada, successfully fabricated and tested a morphing wing demonstrator, equipped with Shape Memory Alloys actuators, during a major morphing wing project financed by the Consortium for Research and Innovation in Aerospace in Quebec called CRIAQ 7.1—Improvement of laminar flow on a research wing. Key objectives were in-flight fuel economy, replacement of conventional control surfaces, reduction of drag to improve range, and reduce flutter risk and vibrations. The experimental model of the morphing wing was a rectangular one, with 0.5 m of chord and 0.9 m of span, and has been manufactured starting from a reference airfoil WTEA-TE1. The upper surface of the model was a flexible skin from a composite material which include a resin matrix, Kevlar fibers, and layers of carbon. Two actuation lines based on the Shape Memory Alloys have been used to morph the skin towards the optimized profile, being controlled in the “open loop” architecture by using both conventional and intelligent control methods. Also, few methodologies for laminar to turbulent flow detection have been experimentally demonstrated, together with a closed-loop morphing wing control method based on the feedback provided by some pressure sensors fixed on the flexible upper surface of the wing [19,20,21,22,23,24].

Recently, aircraft systems are going under a major change where many of the mechanical, pneumatic and hydraulic systems are being replaced by the electrical systems. Boeing 787 and A-380 have comparatively greater number of electrical systems as compared to the conventional aircrafts [25]. Electrical systems come with the benefits that they are lightweight and much quieter and more efficient compared to their hydraulic and pneumatic counterparts. The enabling technology for More Electrical Aircraft (MEA) is power electronics without which it cannot be realized. However, aerospace applications still present challenges to the field of power electronics from the perspective of volume, weight, and size [26]. Advisory council for aeronautics research in Europe has set a target to reduce aircraft CO_2_ emission, fuel optimization, and weight reduction of the aircraft. To achieve this target, aircraft manufacturers are focusing on the development of the MEA technology [27].

In this trend of growth of green aircraft technologies correlated with the replacement of the conventional pneumatic/hydraulic/mechanical actuators with the electrical ones, our team developed a second major morphing wing project on a real aircraft wing equipped with an aileron and morphed with an actuation system equipped with Brushless DC (BLDC) motors. This work was performed under project CRIAQ MDO-505—Morphing architectures and related technologies for wing efficiency improvement.

## 2. Short Description of the Morphing Wing Project

**Figure 1:**
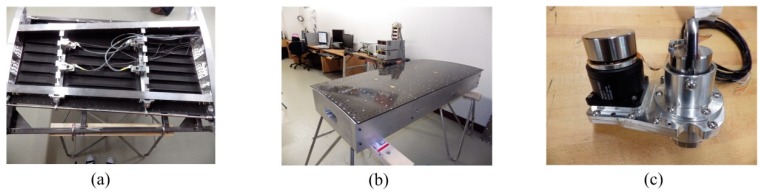
Morphing wing and Brushless DC (BLDC) motor-based actuator.

The project was developed in Canada and Italy, having as its leader the École de Technologie Supérieure (ETS) in Montreal, as Canadian partners, École Polytechnique in Montreal, Institute for Aerospace Research of the National Research Council Canada (IAR-NRC), Bombardier Aerospace, Thales Avionics, and as Italian partners, University of Naples Frederico II, CIRA and Alenia. The project calls for both aerodynamic modeling as well as conceptual demonstration of the morphing principle on real models placed in the wind tunnel, i.e., of a full-scaled portion of a real aircraft wing equipped with an aileron (morphing wing-aileron). The project objective is to obtain a morphing wing-aileron experimental model able to be morphed in a controlled manner and to provide in this way an extension of the laminar airflow region over its upper surface, producing a drag reduction with direct impact on the fuel consumption economy. The physical model developed is shown in Figure 1, where four BLDC motor-based actuators are mounted under the morphing wing along with 32 Kulite pressure sensors used to monitor the transition point.

Our team working at LARCASE is focusing on two main objectives: (a) To sense and monitor the pressure over the flexible skin using pressure sensors, and (b) to develop an actuator control system which can move the laminar to turbulent transition point towards the trailing edge and hence result in large laminar flows.

In the initial aerodynamic studies, some optimized airfoils were computed for 97 flow cases generated as combinations of nineteen values for the angle of attack α (between −3° and +3°), three values for the Mach number *M* (0.15, 0.2 and 0.25), and thirteen values for the aileron deflection angle δ (between −6° and +6°). Computational Fluid Dynamics software in combination with optimization algorithms was used to compute the optimum airfoils shapes. For all of the 97 studied flow cases, the optimum airfoil shape was searched by changing its local thickness in order to extend the laminar region of the upper surface flow [28].

**Figure 2:**
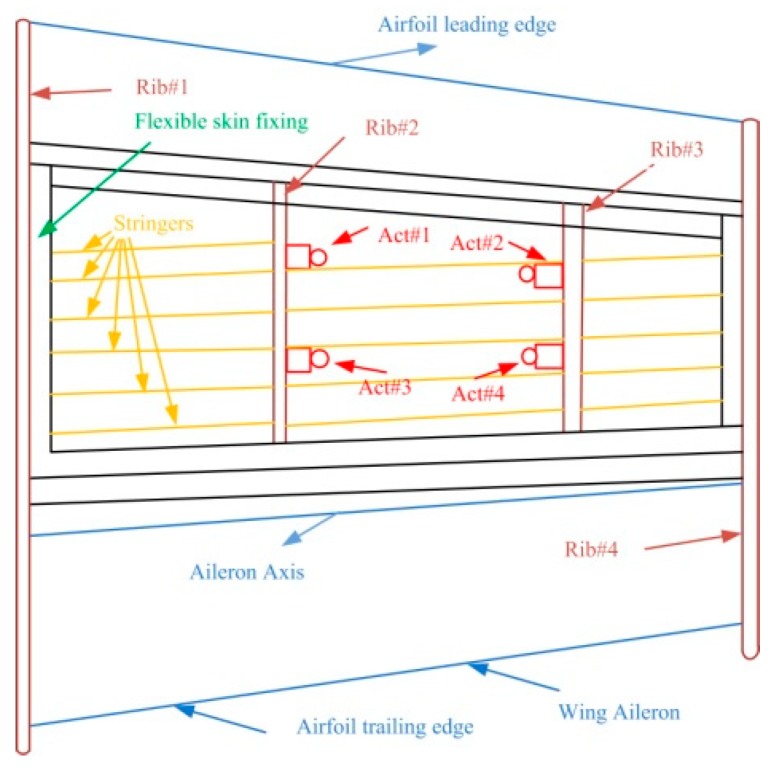
Morphing wing layout.

**Figure 3:**
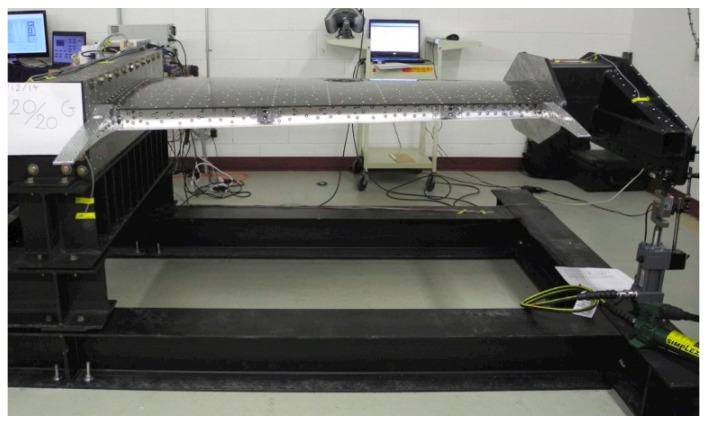
The wing on the bench test at Ecole de Technologie Supérieure (ETS) during 1g structural static test.

At the requirement of the industrial partners involved in the project the wing structure was kept unchanged, similar to the real-life version of the aircraft, except for its upper surface, which was replaced by a flexible skin made from composite material. The model resulted with a 1.5 m span and a 1.5 m root chord, including the aileron, with a taper ratio of 0.72 and a leading-edge sweep of 8°. As shown in Figure 1, the experimental wing model is composed of three parts: (a) unmodified metal structure, (b) flexible upper skin, and (c) actuation system. The flexible skin placed on the upper surface of the wing was delimited by the front and rear spars placed between 20% and 65% of the wing chord. The metal part is composed of four ribs, two of them are in the mid (Rib#2 and Rib#3) and two of them are on the edges (Rib#1 and Rib#4). The actuation system used to morph the wing used four in house developed miniature electrical actuators based on BLDC motors, which performed a direct actuation of the flexible skin. The four identical actuators are placed on the two actuation lines as shown in Figure 2, installed in two sections considered at 37% and 75% of the wing span. Each of the two actuation lines includes two actuators placed at 20% and 65% of the local wing chord. To evaluate the integration of the actuators inside the wing structure and their functionality under a load producing a wing bending, the structural team of the project realized a successful 1g structural static test (Figure 3).

**Figure 4:**
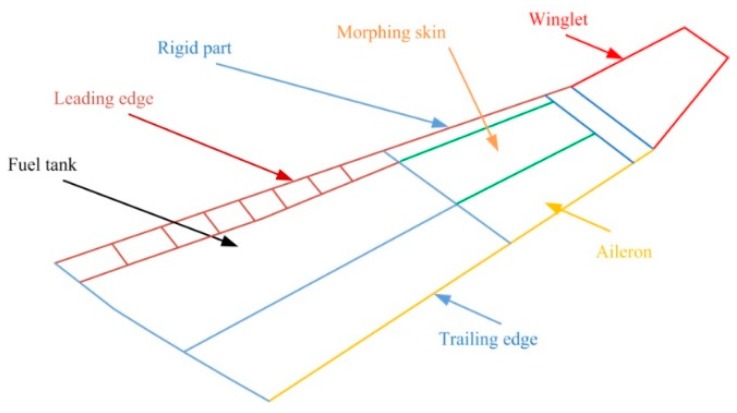
The demonstrator for the morphing wing of the regional aircraft.

Subject wingtip is the demonstrator for the morphing wing of the regional aircraft as shown in Figure 4. The final configuration of the wing-aileron model included a morphing aileron designed and manufactured by the Italian team [29,30].

To evaluate the added value of the morphing technology on our project, the developed experimental model of the morphing wing-aileron system has been tested in the subsonic wind tunnel of the National Research Council of Canada. This testing phase was complex because the team also aimed to validate the results obtained after the numerical optimization of the airfoil shape for all 97 studied flow cases, and to observe the behavior of the experimental model as a whole in various testing conditions that are similar to the ones found in a real flight, both from the point of view of the flow parameters, but also from the point of view of the perturbations, which in this situation were induced by the wind tunnel.

The here presented work refers to the design, numerical simulation and experimental testing of an intelligent control method, based on fuzzy logic technique for a new morphing wing actuation system equipped with BLDC motors. The paper aims also to describe how the experimental morphing wing model has been developed, controlled and tested in order to prove the feasibility of the morphing wing technology for the next generation of aircraft.

## 3. Physical Architecture and Simulink Model of the Controlled Actuator

Over the last decades, BLDC motors have gained much reputation, and they have many applications in aerospace, the automotive industry, medical instruments, and industrial automation. The choice between the Brush DC motor and the BLDC motor really depends on many factors; the difference becomes clearer when the motor has to operate in high temperatures. Due to the absence of carbon brushes, the BLDC has much less wear and tear and more time between the maintenance. Contrastingly, the BLDC motors are electronically commutated and have an improved torque-to-size ratio, which means that they are more useful in the applications where space and weight are critical. The advantages of the BLDC motors, with respect to DC motors, are as follows; better speed ranges than DC motors, improved torque efficiency, less noise, improved efficiency, long operating life, and better weight-to-size ratio [31]. Bearing in mind these reasons, but also the structural particularities of our application, which requires a higher torque-to-size ratio due to the direct actuation in a small space, the team decided to use some actuators based on BLDC motors. Because the market did not offer a convenient solution, our team resorted to manufacturing its own actuators by using some BLDC motors provided by the Maxon Company [32].

**Figure 5:**
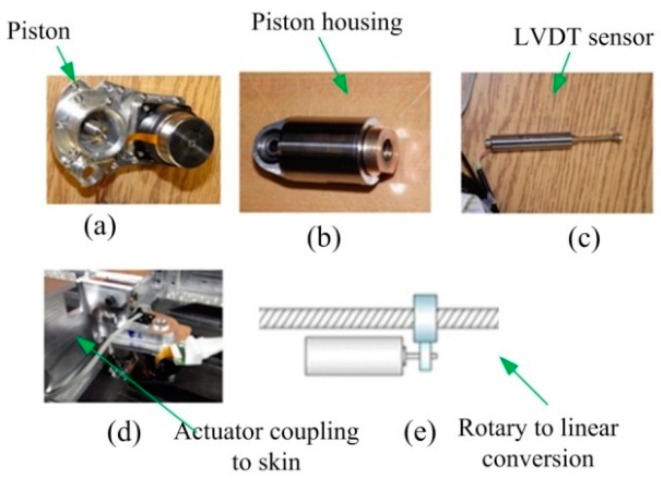
The morphing actuator: (**a**) piston, (**b**) piston housing, (**c**) LVDT sensor, (**d**) actuator coupling with wing skin, and (**e**) principle of rotary to linear conversion.

The novel morphing actuator used in CRIAQ MDO 505 morphing wing project is composed of BLDC motor coupled to the linear actuator through gears (Figure 5). The shaft of the motor is coupled to the gears which in turn are coupled to the screw through gears, in this way the rotational motion of the gear is converted to the linear motion as shown in Figure 5e. Figure 5a,b shows the actuator piston and housing. Figure 5b exposes the mechanical structure, which is mounted on the linear screw and is moved up and down either to push the skin of the wing up or down based on the various flight conditions. Figure 5c depicts the LVDT (Linear Variable Differential Transformer), which is coupled via gears to the linear screw of the actuator to measure the linear displacement travelled by the actuator, and to provide, in this way, the feedback signal for the position control loop. The relationship between the rotational speed of the BLDC motor and the linear screw is such that for each 100 revolutions of motor the linear screw would move 1mm. Figure 5d represents the actuator placement under the morphing skin.

**Figure 6:**
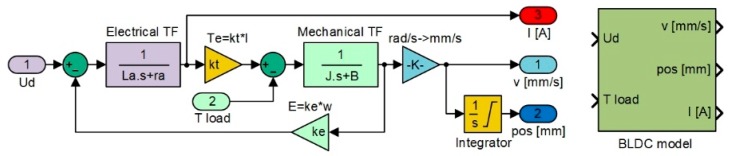
MATLAB/Simulink model of Brushless DC (BLDC) motor.

The control model of the actuators used in CRIAQ MDO 505 project has been presented by the authors of [33]. This section explains the major blocks that were used in the actuator modeling. Major component driving the actuator is the BLDC motor, acquired from Maxon Motor Inc., with nominal voltage of 12 volts, nominal current of 1 ampere, and nominal torque of 25 mN-m [32]. With these characteristics, the motor was considered capable enough to provide the necessary torque to push the skin of the morphing wing. The Matlab/Simulink software model obtained for the linear model of the motor, which was used in the design of the actuator control, as in Figure 6 [33]; it has been called “BLDC model”. The software includes three main parts: the electrical model of the motor, the mechanical model of the motor, and the model of the mechanism converting angular actuation to linear actuation. In the “BLDC model”, the block inputs are the DC bus voltage “Ud” and the load torque “T load”, and provides as outputs the actuation speed “v” (expressed in mm/s), the actuation linear position “pos”, (expressed in mm), and the electrical current “I” (expressed in A).

**Figure 7:**
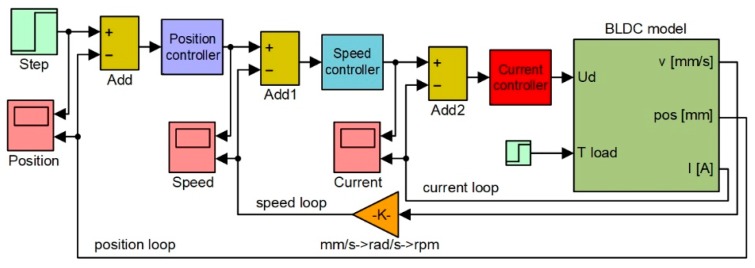
Control system of the morphing actuator based on three control loops.

As shown in Figure 6, the linear position of the actuator can be obtained after incorporating the appropriate gains representing the operation of the gears converting the rotary motion into the linear motion. As the morphing actuator has to push against the load offered by the skin of the wing, there is a need to control the amount of current flowing in the coils of the BLDC motor, which in turn controls the torque produced by the motor. Current control loop is also required for the speed and position control loops as the amount of current decides the speed and torque with which the actuator will acquire the required skin displacement for each flight case. To design the control system of the actuator at the level of its three control loops (for actuation position, actuation speed, and electrical current), the “BLDC model” block was integrated into the model in Figure 7. According to the model in Figure 6, the electrical current results as output of the “Electrical TF” transfer function, and the actuation speed is expressed in rad/s is the output of the “Mechanical TF” transfer function, which is further converted in mm/s, but also provides the actuation linear displacement expressed in mm. All three parameters obtained as outputs of the “BLDC model” block are used as feedbacks for the three control loops of the electrical actuator, as it is presented in Figure 7.

## 4. The Control System Design and Numerical Validation Results

A short literature review can show that the position control of the BLDC motors can be performed in many ways, the simplest one being based on the classical PID controllers with or without all control components inside. Due to the nonlinear character of the system as a whole, generated especially by the complex behavior of the morphed flexible skin interacting with actuators and with the aerodynamic loads appearing in the wind tunnel testing, the team decided to develop an intelligent control variant for the morphing actuation system. Therefore, the system controlling the morphing actuators, which is shown herein, is a nonlinear one, being based on the fuzzy logic technique for all of the three control loops used for each of the four actuators.

Fuzzy controllers are based on fuzzy inference systems (FIS’s), which are composed of several steps. Firstly, the inputs are mapped into appropriate membership functions, following which the IF-THEN logic rules are created. The IF’s are known as “antecedents”, while the THEN’s are known as “consequents”. Based on membership grade, all the rules that are invoked are combined. In the final stage, the combined result from all the rules is converted into a specific output control value.

**Figure 8:**
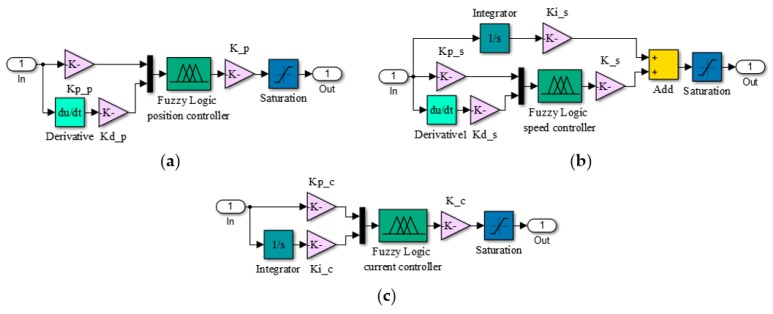
Structures of the controllers used in the three loops: (**a**) position, (**b**) speed, and (**c**) current.

**Figure 9:**
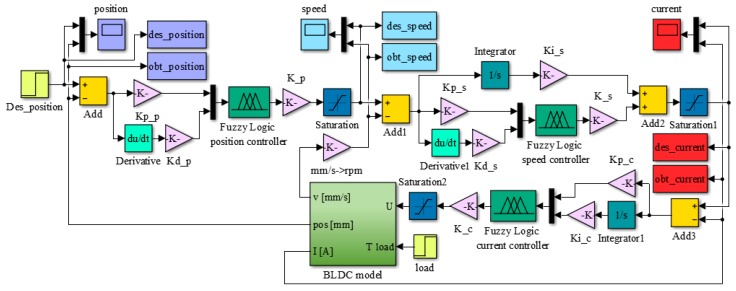
Matlab/Simulink model for the morphing actuator control system.

The numerical simulations achieved in the design phase provided a fuzzy logic Proportional-Derivative architecture for the position control loop, with the schema in Figure 8a; a fuzzy logic Proportional-Integral-Derivative architecture for the speed control loop, with the schema in Figure 8b; and a fuzzy logic Proportional-Integral architecture for the electrical current control loop, with the schema in Figure 8c. The obtained FISs were called “PositionFIS” (for the position controller), “SpeedFIS” (for the speed controller), and “CurrentFIS”, for the electrical current controller. The elements in Figure 8 are Kp_p: proportional gain in position control loop; Kd_p: derivative gain in position control loop; K_p: change in output gain in position control loop; Kp_s: proportional gain in speed control loop; Kd_s: derivative gain in speed control loop; Ki_s: integral gain in speed control loop; K_s: change in output gain in speed control loop; Kp_c: proportional gain in current control loop; Ki_c: integral gain in current control loop; and K_c: change in output gain in current control loop. With the three controllers structures presented in Figure 8, the Matlab/Simulink model for the morphing actuator control system resulted as in Figure 9.

Considering the [−4,4] interval as the universe of discourse for the first input of the “PositionFIS” and [−5 × 10^4^, 5 × 10^4^] interval as universe of discourse its second input, six membership functions (*mf*) were chosen for each of the two inputs ($A_{1}^{1}$ to $A_{1}^{6}$, respectively, $A_{2}^{1}$ to $A_{2}^{6}$). The linguistic terms for both inputs, but also for the output, were NB (negative big), NM (negative medium), NS (negative small), PS (positive small), PM (positive medium), and PB (positive big). The considered shapes for the first input membership functions were *z*-functions (*mf*1), π-functions (*mf*2 to *mf*5, respectively), and *s*-functions (*mf*6), whereas for the second input membership functions shapes was a triangular one.

From the perspective of the “SpeedFIS” fuzzy inference system, [−150,150] interval was chosen as universe of discourse for the first input, and [−1.5 × 10^4^, 1.5 × 10^4^] interval as universe of discourse for its second input. This time, seven membership functions (*mf*) were chosen for each of the two inputs of the FIS, while, from the linguistic terms point of view, for both inputs, but also for the output, a new one has been added (Z (zero)) comparatively with the “PositionFIS”. The considered shapes for the both inputs membership functions were *z*-functions (*mf*1), π-functions (*mf*2 to *mf*6), respectively *s*-functions (*mf*7).

A simplified situation was for the “CurrentFIS” fuzzy inference system, where each of the two inputs were used with two membership functions, with *z*-function (*mf*1), respectively, *s*-function shapes (*mf*2). [−3,3] and [−0.01 0.01] intervals were chosen as universes of discourse for the first input, and for the second input, respectively. In this case, the linguistic terms for both inputs were N (negative) and P (positive), while for the output were N (negative), Z (zero) and P (positive).

An *s*-function shaped membership function can be implemented using a cosine function [22]:

(1) $$s\left(x_{left},\;x_{right},\;x\right)=\{\begin{array}{cc} 0, & \mathrm{if}\;x<x_{left}\\ \frac{1}{2}\left[1+\cos\left(\frac{x-x_{right}}{x_{right}-x_{left}}\pi \right)\right], & \mathrm{if}\;x_{left}\leq x\leq x_{right}\\ 1, & \mathrm{if}\;\;\;x>x_{right} \end{array},$$

a *z*-function shaped membership function is a reflection of a shaped *s*-function [22]:

(2) $$z\left(x_{left},\;x_{right},\;x\right)=\{\begin{array}{cc} 0, & \mathrm{if}\;x<x_{left}\\ \frac{1}{2}\left[1+\cos\left(\frac{x-x_{left}}{x_{right}-x_{left}}\pi \right)\right], & \mathrm{if}\;x_{left}\leq x\leq x_{right}\\ 1, & \mathrm{if}\;\;\;x>x_{right} \end{array},$$

and a π -function shaped membership function is a combination of both functions [22]:

(3) $$\pi \left(x_{left},\;x_{m1},\;x_{m2},\;x_{right},\;x\right)=\min\left[s\left(x_{left1},\;x_{m1},\;x\right),\;\;\;\;z\left(x_{m2},\;x_{right},\;x\right)\right],$$

with the peak flat over the [*x_m_*_1_, *x_m_*_2_] middle interval. *x* is the independent variable on the universe of discourse, *x_left_* is the left breakpoint, and *x_right_* is the right breakpoint [22]. In the other way, the triangular shape can be expressed as follows [34]

(4) $$f_{\Delta }\left(x;a,b,c\right)=\{\begin{array}{cc} 0, & \mathrm{if}\;x\leq a,\\ \frac{x-a}{b-a}, & \mathrm{if}\;a<x<b,\\ \frac{c-x}{c-b}, & \mathrm{if}\;b\leq x<c,\\ 0, & \mathrm{if}\;c\leq x, \end{array}=\max\left[\min\left(\frac{x-a}{b-a},\frac{c-x}{c-b}\right),\;\;0\right].$$

Similarly, *x* is the independent variable on the universe of discourse, while the parameters *a* and *c* locate the feet of the triangle and *b* gives its peak.

In accordance with the expressions given in Equations (1) to (4), the parameters characterizing the membership functions for the first input of the “PositionFIS” and for both inputs of the “CurrentFIS” are given in Table 1, and the parameters characterizing the membership functions for both inputs of the “SpeedFIS” are given in Table 2, whereas the parameters characterizing the membership functions for the second input of “PositionFIS” are listed in Table 3.

The rules for all three fuzzy inference systems involved in the control loops were defined using the Sugeno fuzzy model, which was proposed by Takagi, Sugeno and Kang [35]. According to this model, a fuzzy rule for a two input-single output system can be expressed as follows,

(5) $$“\mathrm{if}\;\;\left(x_{1}\;\mathrm{is}\;A\right)\;\;\mathrm{and}\;\;\left(x_{2}\;\mathrm{is}\;B\right)\;\;\;\;\mathrm{then}\;\;\;\;y=f\left(x_{1},x_{2}\right)”,$$

where *A* and *B* are fuzzy sets in the antecedent, *y* = *f*(*x*_1_, *x*_2_) is a crisp function in the consequent, and *f* is a polynomial function [34,35]. If *f* is a constant, then the Sugeno fuzzy model is a zero-order model. Considering [−3000, 3000] interval as universe of discourse for the “PositionFIS” output and a zero-order Sugeno fuzzy model, the output *mf* were chosen as constants with the values: NB = −3000, NM = −2500, NS = −1000, PS = 1000, PM = 2500, and PB = 3000. Also, for the output of the “SpeedFIS”, the [−120, 120] interval was used as universe of discourse, and the *mf* resulted with the values NB = −120, NM = −60, NS = −30, Z = 0, PS = 30, PM = 60, and PB = 120, whereas for the “CurrentFIS” output, the [−1.5, 1.5] interval was used as universe of discourse, and the *mf* resulted with the values N = −1.5, Z = 0, and P = 1.5.

**Figure 10:**
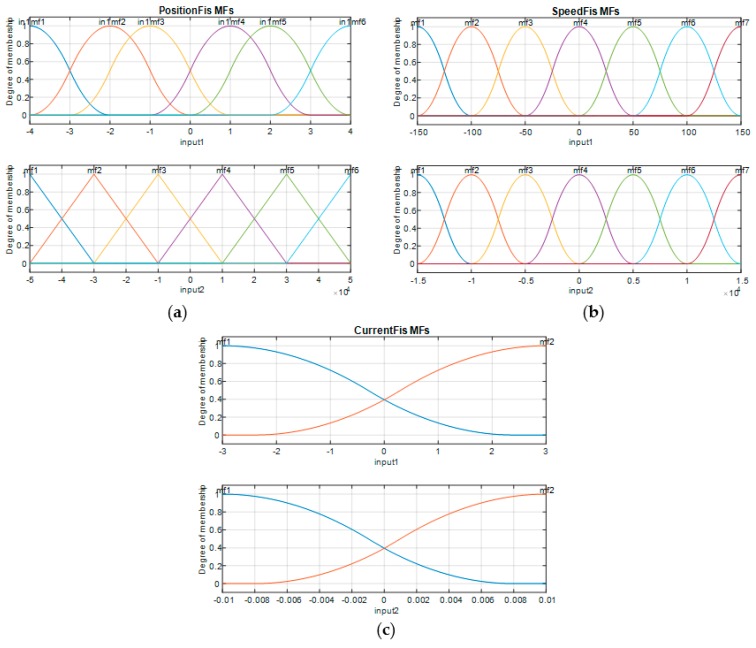
Membership functions for the FISs inputs: (**a**) PositionFIS, (**b**) SpeedFIS, and (**c**) CurrentFIS.

According to the values in Table 1 Table 2 Table 3, the membership functions associated to the inputs of the “PositionFIS”, “SpeedFIS” and “CurrentFIS” are by the forms exposed in Figure 10.

Starting from the inputs’ and output’s membership functions of the three FISs, a set of sec inference rules were obtained for the “PositionFIS”:

(6) $$\begin{array}{cc} \mathrm{Rule}\;\;1: & If\;in1\;is\;A_{1}^{1}\;and\;in2\;is\;A_{2}^{1},\;\;then\;y^{1}\left(in1,\;in2\right)=-3000,\\ \mathrm{Rule}\;\;2: & If\;\;in1\;is\;A_{1}^{2}\;and\;in2\;is\;A_{2}^{2},\;\;then\;y^{2}\left(in1,\;in2\right)=-2500,\\ \mathrm{Rule}\;\;3: & If\;in1\;is\;A_{1}^{3}\;and\;in2\;is\;A_{2}^{3},\;\;then\;y^{3}\left(in1,\;in2\right)=-1000,\\ \mathrm{Rule}\;\;4: & If\;in1\;is\;A_{1}^{4}\;and\;in2\;is\;A_{2}^{4},\;\;then\;y^{4}\left(in1,\;in2\right)=1000,\\ \mathrm{Rule}\;\;5: & If\;in1\;is\;A_{1}^{5}\;and\;in2\;is\;A_{2}^{5},\;\;then\;y^{5}\left(in1,\;in2\right)=2500,\\ \mathrm{Rule}\;\;6: & If\;in1\;is\;A_{1}^{6}\;and\;in2\;is\;A_{2}^{6},\;\;then\;y^{6}\left(in1,\;in2\right)=3000, \end{array}$$

**Figure 11:**
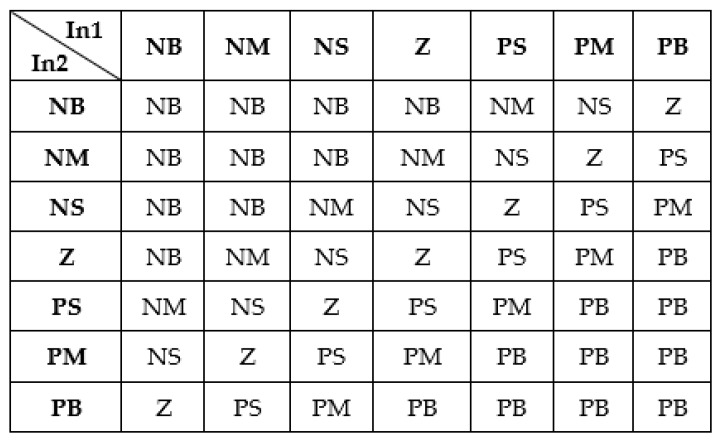
The inference rules for the “SpeedFIS”.

Forty-nine rules for the “SpeedFIS” (Figure 11), and four rules for the “Current FIS”:

(7) $$\begin{array}{cc} \mathrm{Rule}\;\;1: & If\;in1\;is\;A_{1}^{1}\;and\;in2\;is\;A_{2}^{1},\;\;then\;y^{1}\left(in1,\;in2\right)=-1.5,\\ \mathrm{Rule}\;\;2: & If\;\;in1\;is\;A_{1}^{1}\;and\;in2\;is\;A_{2}^{2},\;\;then\;y^{2}\left(in1,\;in2\right)=0,\\ \mathrm{Rule}\;\;3: & If\;in1\;is\;A_{1}^{2}\;and\;in2\;is\;A_{2}^{1},\;\;then\;y^{3}\left(in1,\;in2\right)=0,\\ \mathrm{Rule}\;\;4: & If\;in1\;is\;A_{1}^{2}\;and\;in2\;is\;A_{2}^{2},\;\;then\;y^{4}\left(in1,\;in2\right)=1.5, \end{array}$$

**Figure 12:**
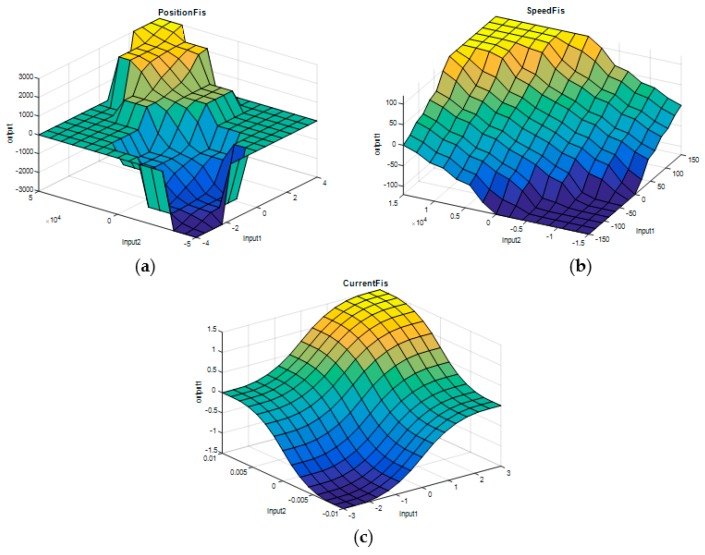
The fuzzy control surfaces for the three FISs: (**a**) PositionFIS, (**b**) SpeedFIS, and (**c**) CurrentFIS.

Starting from the characteristics previously specified, for the three fuzzy inference systems, the control surfaces have been obtained with the shapes presented in Figure 12.

**Figure 13:**
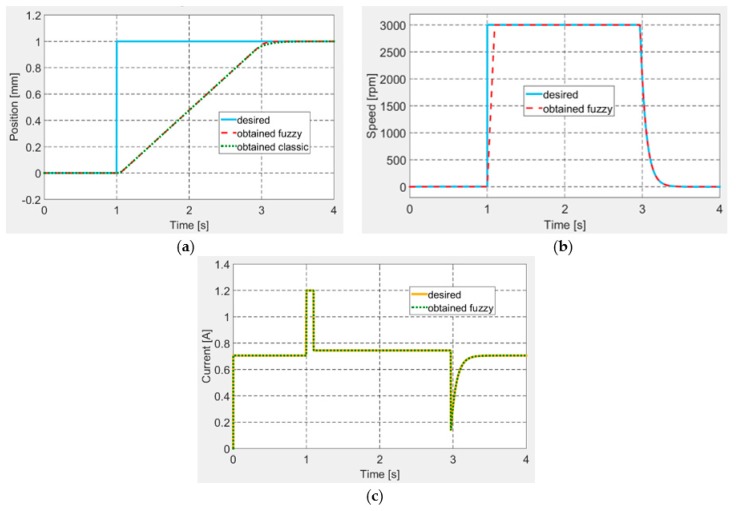
The control results for a step input as desired position: (**a**) position, (**b**) speed, and (**c**) current.

Following a tuning procedure, the best values of the controllers’ gains were established and further used in the control system Matlab/Simulink model, to test it through numerical simulation by using various signals as desired inputs. In a first numerical simulation test, a step input was applied as desired signal for the actuation position. The obtained results are shown in Figure 13 for all of the three controlled parameters: position, speed and electrical current. It can be easily observed that the designed control system worked very well in all of the three control loops. Moreover, in Figure 13a, which depicts the controlled actuation position, are presented together the results obtained by using the here design controller, but also the curve obtained if it is used a classical variant for the control system, designed also by our team research team [33]. A short analysis of the two answers proves that the fuzzy logic-based control system provides a small settling time comparatively with the classical control system.

**Figure 14:**
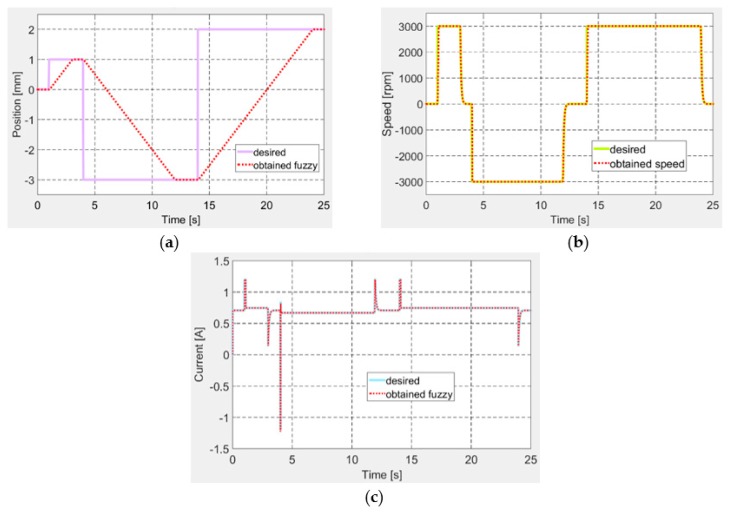
Control for successive steps signal as desired position: (**a**) position, (**b**) speed, and (**c**) current.

Another important test required for the actuator to follow a desired position signal under the form of successive steps, so as to test the ability of the actuation system to switch between positive and negative positions, similarly with the actuation situations, resulted from the numerical optimization performed for the 97 flow cases. The results for this test are exposed in Figure 14, confirming once again a very good operation of the control system in all of the three control loops.

Once completed the design and testing through numerical simulation for the control system, the research team sent the morphing wing project at the next level, performing the integration of all components in the experimental model and preparing it for the wind tunnel tests.

## 5. Wind Tunnel Experimental Testing of the Wing-Aileron Morphing System

To evaluate the impact of the morphing technology on our experimental model, the set of the 97 flow cases was studied both from numerical and experimental points of view. During the experimental evaluation performed in the wind tunnel testing facility of the Canadian National Research Council in Ottawa, the airflow over the upper surface was monitored for all studied flow cases by using two techniques: (1) the real-time processing of the pressure data in a section along the wing chord, which were collected by using 32 Kulite pressure sensors; (2) the Infrared (IR) thermography based on a Jenoptik camera, which provided captions for the airflow over the entire upper surface of the wing. Besides the real-time monitoring, a postprocessing phase of the pressure data has been done; 20 kSamples/s was the rate for the pressure data recording in all of the 32 detection channels, both for original (un-morphed) and optimized (morphed) airfoils tested in the 97 flow cases. The main instruments in both pressure data processing phases were the fast Fourier transform (FFT) and the standard deviation (STD), which, together, and based on different scientific arguments, served at the estimation of the laminar-to-turbulent airflow transition point position in the monitored section (2D estimation of this position). On the other way, the IR captions validated the technique based on the pressure data processing, but, more important, having in mind that the tested morphing wing has a complex structure, allowed the researchers to evaluate the global aerodynamic gain produced by the morphing technology. This was possible because the IR technique provided captions with the whole upper surface of the wing, which facilitated the estimation of the position for the laminar-to-turbulent airflow transition region along the whole wing span (3D estimation of this position).

**Figure 15:**
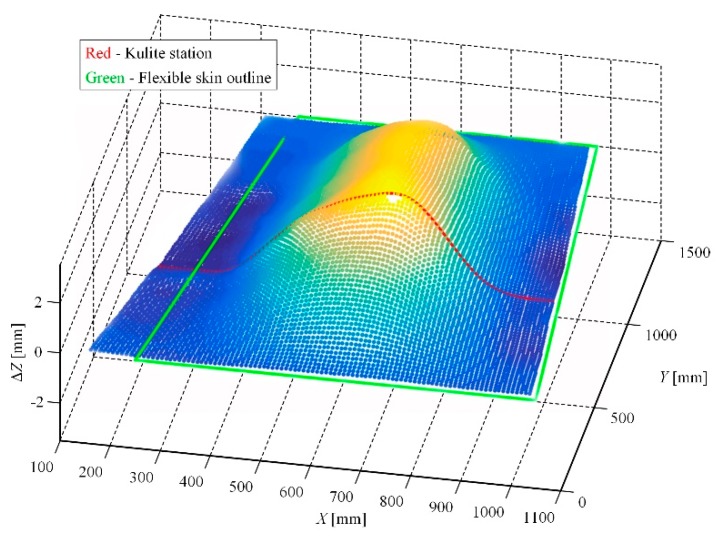
Laser scan of the morphed wing shape for the *M* = 0.2, α = 2°, and δ = 4° flow case.

As a preparatory step for the wind tunnel tests, some calibrations were performed, both in the LARCASE laboratory with no airflow, but also when the model was fixed in the wind tunnel testing room. To evaluate the morphed shape of the wing, a laser scanning was realized in the LARCASE laboratory with the actuation system controlled for all of the 97 studied flow cases. For example, in the flow case characterized by *M* = 0.2, α = 2°, and δ = 4°, the laser scanning of the wing upper surface provided the picture shown in Figure 15; there is figured just the scan of the wing, without the aileron. The actuation distances obtained from the numerical optimization of this flow case were 2.89 mm, 2.95 mm, 2.71 mm, and 3.44 mm. Also, during the calibrations procedures performed in the LARCASE laboratory an evaluation of the actuators loads was realized. The evaluation consisted in the measurement of the forces acting on the actuators when only one of them was actuated, and the others were kept in the positions characterizing the reference airfoil of the wing. For each one, the actuation distance was modified between −3 mm and 3 mm. Each time, the extreme values of the forces were obtained for the energized actuator. Thus, when the first actuator was used to morph the flexible skin between −3 mm and 3 mm the force loading it varied between 1300 N and −2000 N. When the second actuator was energized, the measured values of the force loading it varied between 1720 N and −1900 N, whereas, in the same actuation conditions, the forces loading the third and the fourth actuators varied between 2400 N and −2200 N, respectively, between 1720 N and −1900 N.

**Figure 16:**
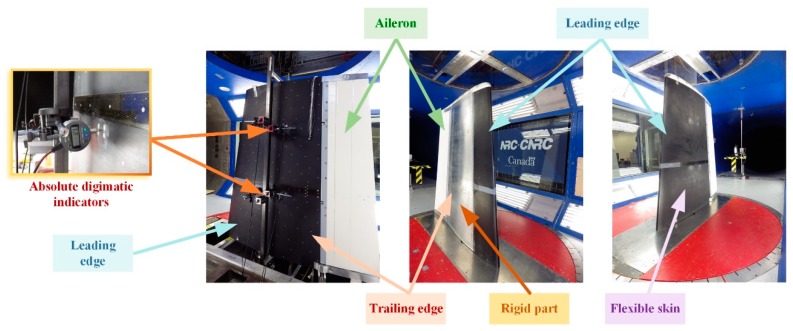
Morphing wing-aileron experimental model in the Institute for Aerospace Research of the National Research Council Canada (IAR-NRC) wind tunnel testing room.

**Figure 17:**
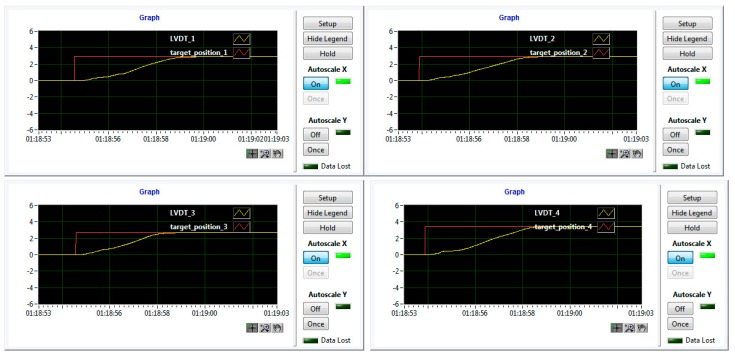
Actuators real-time monitoring for the *M* = 0.2, α = 2°, and δ = 4° flow case with the wing morphed.

During the wind tunnel tests, the morphing wing-aileron experimental model has been fixed in vertical position in the IAR-NRC wind tunnel testing room (Figure 16). Once fixed on the testing position, the model has been subjected to a new set of calibrations, this time by using some absolute digimatic indicators. The estimated corrections completed the final version of the application software, working with the control system during the wind tunnel tests. The software component developed by the team included also a Graphic User Interface (GUI) containing some graphical windows and buttons that allowed the users to monitor and control the experimental model during testing, but also to visualize what is happening at the level of the laminar to turbulent transition point position in the Kulite pressure sensors station (2D estimation of the transition position). Figure 17 shows a part of the GUI monitoring in real-time the controlled actuation positions for all of the four actuators in the morphed configuration of the wing, for the flow case characterized by *M* = 0.2, α = 2°, and δ = 4° conditions.

**Figure 18:**
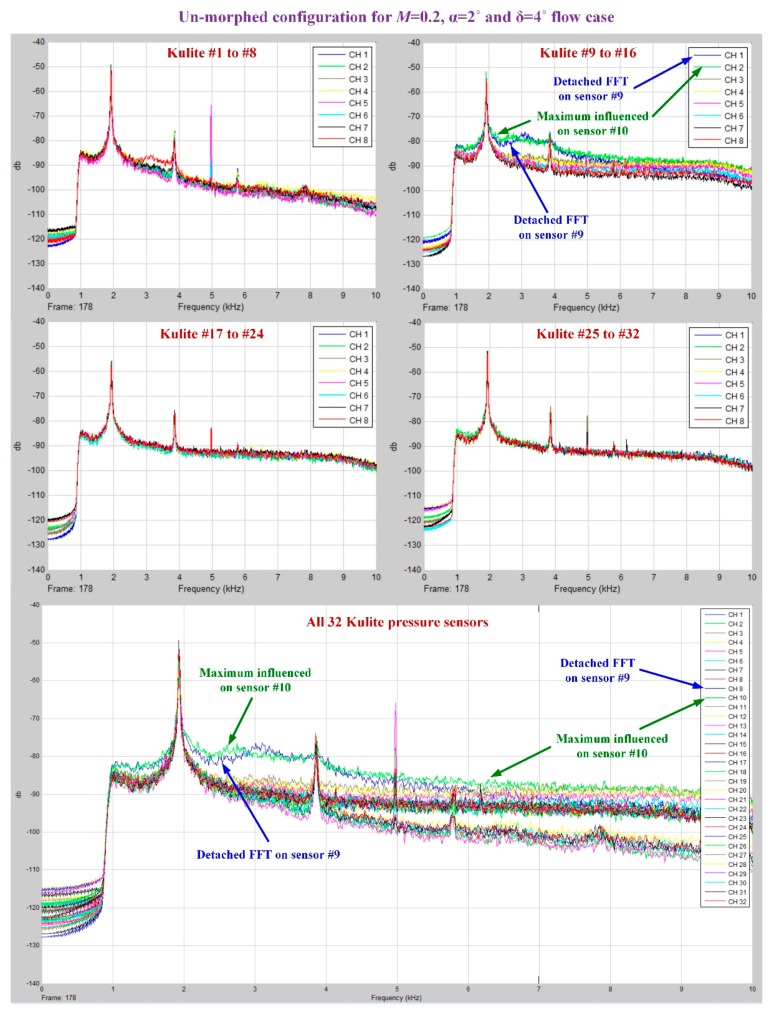
Fast Fourier transform (FFT) results for the wing un-morphed configuration in *M* = 0.2, α = 2°, and δ = 4° flow conditions.

**Figure 19:**
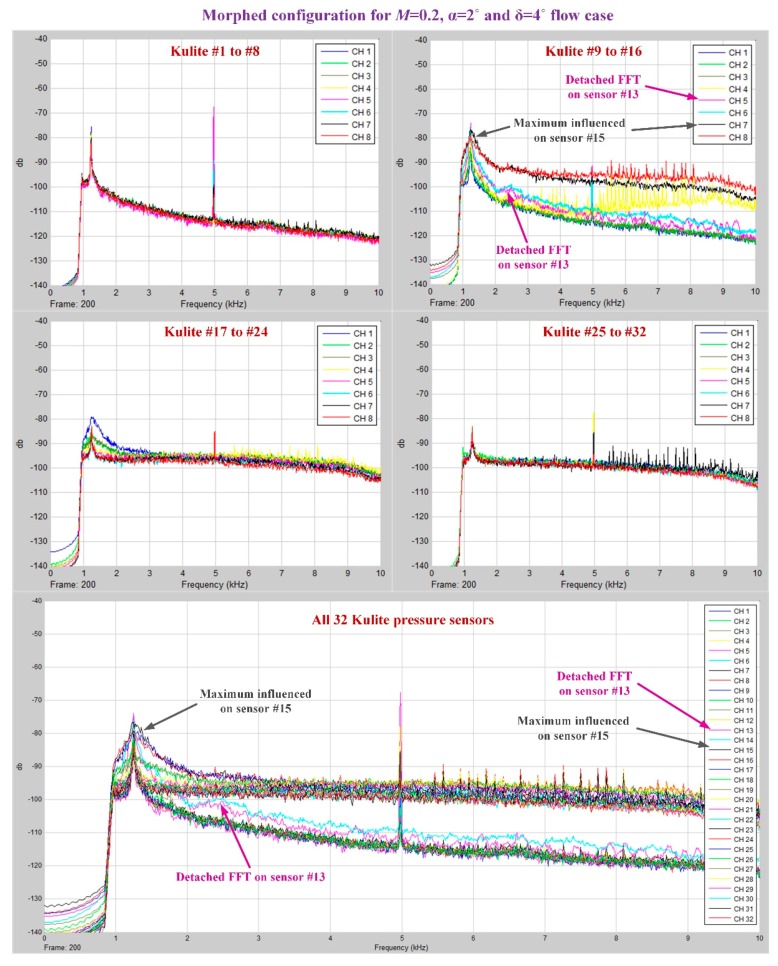
FFT results for the wing morphed configuration in *M* = 0.2, α = 2°, and δ = 4° flow conditions.

**Figure 20:**
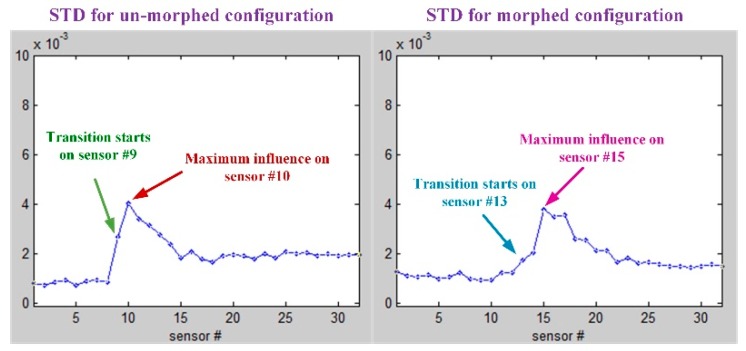
Standard deviation (STD) results for *M* = 0.2, α = 2°, and δ = 4° flow conditions.

As was already mentioned, in the Kulite sensors station it was performed a 2D evaluation of the airflow laminar to turbulent transition point position based on the pressure data processing by using the fast Fourier transform (FFT) and the standard deviation (STD). Figure 18; Figure 19 describe the FFT characteristics obtained for un-morphed and morphed airfoils when the flow conditions were *M* = 0.2, α = 2° and δ = 4°, whereas Figure 20 presents the results for the STD evaluation for both airfoils in the same flow case. It can be observed that FFTs and STDs curves suggest the same regions for the transition location in the un-morphed configuration, but also in the morphed one. Therefore, for the un-morphed airfoil both FFT and STD indicated that the passing from laminar to turbulence was made somewhere in the area of the 10th Kulite sensor, i.e., at 42.45% of the wing chord, whereas for the morphed airfoil was made somewhere in the area of the 15th Kulite sensor, i.e., at 50.04% of the wing chord.

**Figure 21:**
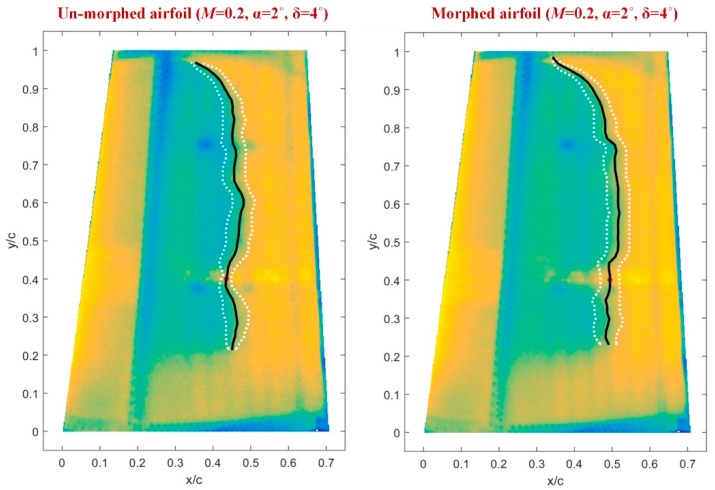
The infrared thermography results for *M* = 0.2, α = 2°, and δ = 4° flow conditions.

The second method facilitating the estimation of the position for the laminar-to-turbulent airflow transition region, but this time along the whole wing span, is infrared (IR) thermography (3D estimation of the transition position). The IR results for *M* = 0.2, α = 2°, and δ = 4° flow conditions are shown in Figure 21, for both un-morphed and morphed configurations of the wing. In the two pictures from Figure 21, the doted white lines mark the transition fronts obtained after the next operations were performed with the IR captions: image decimation, detection using gradient image analysis, filtering, and thresholding. In the same pictures, the black lines mark the mean transition between the two transition fronts, and the red dot highlights the transition point position for the Kulites section evaluated with IR technique. The IR evaluation for *M* = 0.2, α = 2°, and δ = 4° flow conditions provided an average value for the laminar to turbulent transition point position along the wing span (the average of all points on the black line) of 43.9767% from the wing chord for un-morphed configuration, and of 47.9681% from the wing chord for morphed configuration. Also, the position of the red dots, i.e., the transition point position for the Kulites station evaluated with IR technique, was found at 42.6925% from the wing chord for un-morphed configuration, and at 49.3381% from the wing chord for morphed configuration, validating in this way the results obtained with the technique based on the FFT and STD evaluation.

Therefore, the IR technique proved an improvement (the difference between morphed and un-morphed configuration) of the 47.9681% − 43.9767% = 3.9914% from the wing chord, related to the average value for the laminar to turbulent transition point position along the wing span, and of 49.3381% − 42.6925% = 6.6456% from the wing chord, related to the transition point position for the Kulites station. At the same time, the technique based on the FFT and STD evaluation showed an improvement of 50.04% − 42.45% = 7.59% of the wing chord, related to the transition point position for the Kulites station.

The final analysis of the FFT, SDT, and IR results for both un-morphed and morphed airfoils revealed that the morphing technology improved the average position of the laminar to turbulent flow transition over the whole wing, with more than 2.5% of the wing chord for the great majority of the studied flow cases.

## 6. Conclusions

The paper exposed a part of the work done in a major morphing wing international research project developed as a collaboration between Canadian and Italian partners from industry, research, and academic fields. The project intended to demonstrate the feasibility of the morphing wing technology for the next generation of aircraft by developing a morphing wing-aileron experimental model, starting from a full-scaled portion of a real aircraft wing, and testing it in the wind tunnel.

The information presented herein, as results of the research project, proved that the team obtained an experimental wing model able to be morphed in a controlled manner and to provide in this way an extension of the laminar airflow region over its upper surface, producing a drag reduction with direct impact on the fuel consumption economy. The paper highlighted the results obtained for the design, numerical simulation and wind tunnel experimental testing of a fuzzy logic-based control variant for the morphing wing tip actuation system, but also the aerodynamic gain produced by the morphing technology on our experimental model.

The control system structure for the morphing actuation system included three loops, the designed fuzzy logic-based control variant leading to the next configuration: a Proportional-Derivative architecture for the position control loop, a Proportional-Integral-Derivative architecture for the speed control loop, and a Proportional-Integral architecture for the electrical current control loop. All tests demonstrated a very good operation of the control system in all of the three control loops.

From another perspective, the wind tunnel testing of the integrated morphing wing-aileron experimental model showed promising aerodynamic gain for the morphed configuration in front of the un-morphed one. A set of the 97 flow cases were studied by the research team, both from numerical and experimental points of view, to estimate the added value of the morphing technology. Also, during the wind tunnel testing, the team used two techniques to monitor the airflow over the upper surface and to evaluate in this way the position of the laminar-to-turbulent airflow transition region: (1) the processing of the pressure data for a section along the wing chord, which were collected using 32 Kulite pressure sensors (2D estimation of the transition position), and (2) the infrared (IR) thermography (3D estimation of the transition position).

The analyze of the results for the estimation of the transition position, for both un-morphed and morphed airfoils, revealed that the morphing technology improved the average position of the laminar to turbulent flow transition over the whole wing, with more than 2.5% of the wing chord for the great majority of the 97 studied flow cases.

## Figures and Tables

**Table 1:** Parameters of the *mf* for the “PositionFIS” first input and for the “CurrentFIS” both inputs.

	PositionFIS/Input1						CurrentFIS/Input1		CurrentFIS/Input2	
Param.	*mf*1	*mf*2	*mf*3	*mf*4	*mf*5	*mf*6	*mf*1	*mf*2	*mf*1	*mf*2
*x_left_*	−4	−4	−3	−1	0	2	−3	−2.4	−0.01	−0.008
*x_m_*_1_		−2	−1	1	2					
*x_m_*_2_		−2	−1	1	2					
*x_right_*	−2	0	1	3	4	4	2.4	3	0.008	0.01

**Table 2:** Parameters of the *mf* for the both inputs of the “SpeedFIS”.

	SpeedFIS/Input1							SpeedFIS/Input2						
Param.	*mf*1	*mf*2	*mf*3	*mf*4	*mf*5	*mf*6	*mf*7	*mf*1	*mf*2	*mf*3	*mf*4	*mf*5	*mf*6	*mf*7
*x_left_*	−150	−150	−100	−50	0	50	100	−1.5 × 10^4^	−1.5 × 10^4^	−1 × 10^4^	−5 × 10^3^	0	5 × 10^3^	1 × 10^4^
*x_m_*_1_		−100	−50	0	50	100			−1 × 10^4^	−5 × 10^3^	0	5 × 10^3^	1 × 10^4^	
*x_m_*_2_		−100	−50	0	50	100			−1 × 10^4^	−5 × 10^3^	0	5 × 10^3^	1 × 10^4^	
*x_right_*	−100	−50	0	50	100	150	150	−1 × 10^4^	−5 × 10^3^	0	5 × 10^3^	1 × 10^4^	1.5 × 10^4^	1.5 × 10^4^

**Table 3:** Parameters of the *mf* for the second input of the “PositionFIS”.

	PositionFIS/Input2					
Param.	*mf*1	*mf*2	*mf*3	*mf*4	*mf*5	*mf*6
*a*	−7 × 10^4^	−5 × 10^4^	−3 × 10^4^	−1 × 10^4^	1 × 10^4^	3 × 10^4^
*b*	−5 × 10^4^	−3 × 10^4^	−1 × 10^4^	1 × 10^4^	3 × 10^4^	5 × 10^4^
*c*	−3 × 10^4^	−1 × 10^4^	1 × 10^4^	3 × 10^4^	5 × 10^4^	7 × 10^4^

## Nomenclature

*A*, *B*	fuzzy sets in the antecedent
$A_{i}^{j}$	inputs’ membership functions
*a*, *c*	parameters locating the feet of the triangle in triangular membership functions
*b*	parameter giving the peak of the triangle in triangular membership functions
*f*	polynomial function
K_c	change in output gain in current control loop
K_p	change in output gain in position control loop
K_s	change in output gain in speed control loop
Kd_p	derivative gain in position control loop
Kd_s	derivative gain in speed control loop
Ki_c	integral gain in current control loop
Ki_s	integral gain in speed control loop
Kp_c	proportional gain in current control loop
Kp_p	proportional gain in position control loop
Kp_s	proportional gain in speed control loop
*M*	Mach number
*s*	*s*-function shaped membership function
*x*	the independent variable on the universe of discourse
*x_left_*	the left breakpoint
*x_mi_*	parameters delimitating the middle interval for a π -function
*x_right_*	the right breakpoint
*y*	crisp function in the consequent
*z*	*z*-function shaped membership function
BLDC	Brushless Direct Current
FIS	Fuzzy Inference System
FFT	Fast Fourier Transform
GUI	Graphic User Interface
IR	Infrared
MEA	More Electrical Aircraft
*mf*	membership function
N	negative
NB	negative big
NM	negative medium
NS	negative small
P	positive
PB	positive big
PD	Proportional-Derivative
PI	Proportional-Integral
PID	Proportional-Integral-Derivative
PM	positive medium
PS	positive small
SMA	Shape Memory Alloy
STD	Standard Deviation
Z	zero
α	angle of attack
δ	aileron deflection angle
π	π-function shaped membership function
